# Does Faeces Excreted by Moxidectin-Treated Sheep Impact Coprophagous Insects and the Activity of Soil Microbiota in Subtropical Pastures?

**DOI:** 10.1155/2024/1960065

**Published:** 2024-02-07

**Authors:** Susana Gilaverte Hentz, Felix Guillermo Reyes Reyes, Glaciela Kaschuk, Leandro Bittencourt de Oliveira, Maria Angela Machado Fernandes, Alda Lúcia Gomes Monteiro

**Affiliations:** ^1^Sheep and Goat Production and Research Center, Federal University of Paraná, Rua dos Funcionários, 1540, CEP 80035-050, Curitiba, PR, Brazil; ^2^Department of Food Science and Nutrition, State University of Campinas, Rua Monteiro Lobato, 80, CEP 13083-862, Campinas, SP, Brazil; ^3^Department of Soil and Agricultural Engineering, Federal University of Paraná, Rua dos Funcionários, 1540, CEP 80035-050, Curitiba, PR, Brazil; ^4^Department of Crop Science and Plant Protection, Federal University of Paraná, Rua dos Funcionários, 1540, CEP 80035-050, Curitiba, PR, Brazil

## Abstract

Moxidectin (MOX) is used to control helminth parasites in ruminant livestock. It is released through feces and remains in the environment for a long period. This study aimed to evaluate the impact of faeces excreted by moxidectin-treated sheep on soil biodiversity (coprophagous insects, soil microbial biomass, and activity) to establish environment-related guidelines regarding the use of MOX in sheep livestock. The study consisted of two experiments. In the first one, faeces from MOX-treated (subcutaneous dose of 0.2 mg·kg^−1^ body weight) and nontreated rams were placed on an animal-free pasture field, protected or not against rain, for 88 days. Then, coprophagous insects were captured, identified, and counted, and faeces degradation was evaluated by measuring dry weight and carbon (C) and nitrogen (N) contents over time. Diptera, Hymenoptera, Isoptera, and Coleoptera were equally encountered in faeces from MOX-treated and nontreated animals. Faecal boluses of MOX-treated animals (with higher N content) not protected against rain degraded faster than faecal boluses of nontreated animals (with lower N content). In the second experiment, faeces from nontreated animals were amended with increasing amounts of MOX (75 to 3,000 ng·kg^−1^ faeces), mixed with soil samples from animal-free pasture (1.9 to 75 ng·kg^−1^ soil), and incubated in a greenhouse for 28 days. Increasing concentrations of MOX did not prevent the growth of cultivable bacteria, actinobacteria, or fungi in culture media. However, even the lower MOX concentration (1.9 ng·kg^−1^ soil) abruptly decreased soil microbial biomass, basal respiration, and N mineralization. Thus, the results indicate that faeces excreted from sheep treated with MOX under the experimental conditions of this study are not harmful to the coprophagous insects. However, adding MOX to faeces from drug-free sheep had a negative impact on soil microbial activity and biomass.

## 1. Introduction

Brazil counted approximately 150 million heads of ruminant livestock (cattle, sheep, goats, and buffaloes) in 2019, accounting for 18% of livestock production in the world[1]. In 2020, Brazil spent approximately US$ 1.5 billion on veterinary drugs, of which 27% were used to control gastrointestinal helminth parasites [2]. Helminth's parasitism decreases dry matter intake, organic matter digestibility, and body weight gains of ruminant livestock, whereas the use of anthelmintics improves animal welfare and growth [3, 4]. Although there are methods to selectively control helminths in sheep production systems, as validated by Kaplan et al. [5], excessive or inadequate anthelmintic applications are often used in livestock [6], probably because of ease and trust in their efficiency.

One distinctive group of anthelmintics is the macrocyclic lactones (ML) that act against arthropods and gastrointestinal nematodes [7] and account for approximately 80% of the veterinary market [8]. Manning et al. [9] noted that MLs are not fully metabolized by ruminants, and their active ingredients are excreted in dung. Moxidectin (MOX) is a milbemycin drug that belongs to the macrocyclic lactones. It accumulates in the animal body, stored mainly in the liver and adipose tissue [7], and it is excreted in the faeces for a long period [10]. The fact that macrocyclic lactones are relatively lipophilic could be a contributing factor to their persistence in the environment, becoming an environmental contaminant, particularly in soil. Faecal excretion is the main route of elimination, representing 50 to 90% of the administered dose, whereas urine excretion is less than 1% of the same dose [11]. Hentz et al. [10] reported that MOX had prolonged persistence in sheep faeces, up to 42 days after subcutaneous application of the drug. When the faeces containing MOX are mixed with the soil, it tends to be strongly bound to the organic matter of the faeces and may persist in the soil minerals and organic matter for several months or possibly for years, incurring risks of contaminating soil and water bodies [12, 13]. Moreover, the permanence of MOX in the soil may impact soil biodiversity and thus affect important soil functions.

The literature on the effects of moxidectin (MOX) on biodiversity is controversial. MOX can be less harmful than ivermectin [14], doramectin, eprinomectin, and ivermectin [15]. Nonetheless, it may negatively impact the abundance of coprophagous insects [16, 17]. MOX has been considered to pose a low risk to invertebrates [9]. However, like other ML endectocides, MOX varies in spectrum and toxicity to arthropod species in the environment [8]. In addition, most studies on the impact of anthelmintics on the environment have been conducted in the Northern Hemisphere. In the Southern Hemisphere, studies in Argentina have shown negative effects of ivermectin, doramectin, and selamectin on the colonization, dispersal, and number of insects in the faecal bolus of cattle exposed to the environment [18–20]; Tovar et al., [21, 22], but moxidectin was not studied. Thus, there is an unfilled gap on the effect of moxidectin residue in faeces on pasture and the present study intends to contribute with information for a better understanding of this phenomenon.

Regarding microorganisms, Lim et al. [23] found that selamectin inhibited the growth of mycobacteria in agar and liquid cultures, even though avermectins are considered effective against helminths, insects, and arachnids, but inactive against annelids, protozoa, bacteria, and fungi [24–26]. On the other hand, few studies have investigated the effect of avermectins on soil microorganisms, which are responsible for several ecosystem soil services. Additionally, microbiological attributes, such as microbial biomass and basal respiration, can indicate the impact of these substances on the environment [27, 28].

Prichard and Geary [8] reported that MOX generally shows low ecotoxicity and emphasized that this drug is suitable for use in high doses and in long-acting formulations, with less risk of developing antiparasitic resistance than avermectins. Thus, considering this perspective and the indiscriminate use of antiparasitic drugs that commonly occurs in livestock, especially in sheep farming systems, concern with environmental health related to the faeces excreted by MOX-treated sheep is pertinent. The hypothesis of this study is that MOX excreted in the faeces of treated sheep drastically decreases the number of decomposing insects and the soil microbiological activity.

Therefore, this study was performed to assess the impact of faeces excreted by MOX-treated sheep on coprophagous insects and the concentration of MOX in sheep faeces on soil microbial biomass and activity to establish environmental guidelines regarding the use of MOX in sheep livestock.

## 2. Materials and Methods

Two studies were conducted sequentially. In the first, faeces from MOX-treated rams were placed in a pasture field experiment, and in the second, MOX was applied directly to drug-free faeces and incubated in soil under greenhouse conditions.

### 2.1. Pasture Field Experiment

#### 2.1.1. Field Experimental Conditions

**Figure 1:**
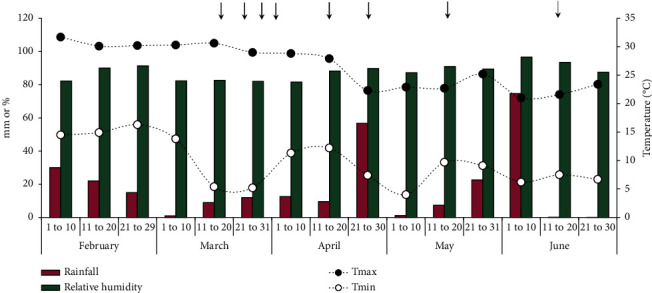
Rainfall (mm), relative humidity (%), and temperature (°C; maximum and minimum) during the experimental period, March to June 2012, near the pasture field experiment in Pinhais, PR, Brazil.

The field experiment was carried out in the pasture field of the Sheep and Goat Production and Research Center (LAPOC) at the Federal University of Paraná, Pinhais, PR, Brazil, during 2012, located at coordinates 25°24′S and 49°07′W at an altitude of 900 m. The climate of the region is classified as Cfb, humid subtropical [29]. The SIMEPAR (Meteorological Systems of Paraná, Pinhais, Brazil) station recorded rainfall, relative humidity, and temperatures during the experiment (Figure 1). The soil, classified as Oxisol under the USDA Soil Taxonomy System [30], contained 454, 46, and 500 g·kg^−1^ of sand, silt, and clay, respectively. The soil had the following chemical characteristics at the beginning of the field experiment: pH-CaCl_2_: 5.0; pH-SMP: 5.9; H + Al: 5.40 cmol_c_·dm^−3^; available nutrients: Ca: 4.6 cmol_c_·dm^−3^; Mg: 2.5 cmol_c_·dm^−3^; P: 4.8 mg·dm^−3^; K: 43.2 mg·dm^−3^; total organic carbon (C): 21.2 g·dm^−3^; organic matter: 3.65%; sum of bases 7.2 cmol_c_·dm^−3^; and saturation of bases: 57%. The pasture was mainly composed of Tifton 85 (*Cynodon* spp.) and ryegrass (*Lolium multiflorum* Lam.). The pasture was kept without animals for more than six months before the start of the experiment.

#### 2.1.2. Treatment of Animals with Moxidectin

Suffolk and White Dorper rams weighing 76.4 ± 34.2 kg and producing an average of 2.69 ± 0.94 kg of fresh faeces per animal were kept in a slatted sheepfold without any drug treatment for 8 months. The animals were fed a diet containing 44.3% concentrate (14% crude protein) and 55.7% ryegrass hay fed *ad libitum*, according to requirements by the NRC [31]. Eight animals received a single subcutaneous MOX dose of 0.2 mg·kg^−1^ body weight (Cydectin® 1% w/v injectable solution (Zoetis, Brazil) was used as the source of MOX). Five animals were not treated with MOX (control group). Thirty-six hours after the application of MOX, faecal collection harnesses were fitted to both MOX-treated and nontreated animals to collect the faeces every 12 h, from the 24th to the 60th h after the application of MOX to animals. The faeces from each animal were collected, homogenized, and stored at 4°C. Then, they were taken to the field to assess the persistence of MOX in the environment. A previous study under similar conditions showed that the concentration of MOX in excreted faeces from treated animals after 88 days of treatment was 30–35 ng·g^−1^ of dry faeces [10]. This study was conducted in accordance with the ethics requirements and approved by the Ethics Committee on Animal Use of the Department of Agricultural Science at the Federal University of Paraná, Brazil (Protocol No. 055/2011).

#### 2.1.3. Experimental Design

The field experiment was arranged in a 2 × 2 factorial design, where the first factor was the MOX treatment (i.e., faeces from MOX-treated and nontreated animals), and the second factor was the field design (with or without protection against rain). The treatments were distributed under a completely randomized block, with four replications and eight sampling times (0, 4, 8, 12, 24, 36, 60, and 88 days of exposure of faeces to the environment). Thus, eight faecal boluses weighing 200 g (67 ± 3.5 g dry matter of faeces) per replicate and per treatment were placed in the pasture field at distances of 2 m between treatments within blocks, 0.5 m between days of exposure, and 8 m between the blocks.

#### 2.1.4. Sampling of Insects

The order Diptera was caught with a sweep net twice a day until the 10th day, on alternate days until the 20th day, every 4 days until the 40th day, and every 8 days until the 88th experimental day.

Pitfall traps were used to collect the orders Diptera, Hymenoptera, and Coleoptera in the first 10 days, with three 500 mL pitfall traps. At the beginning of the experiment (day 0), 15 g of fresh faeces from the MOX-treated and nontreated animals was placed in each pitfall trap and hung by a fine wire attached to the container on two opposite sides. The rain protection formed by an aluminum plate supported by wooden sticks was placed above the pitfall traps.

The direct catch of the orders Coleoptera and Hymenoptera was performed at 4, 8, 12, 24, 36, 60, and 88 days of faecal exposure in the environment.

#### 2.1.5. Faecal Degradation in the Field

Entire faecal boluses were manually collected after 0, 4, 8, 12, 24, 36, 60, and 88 days of exposure in the pasture field. The faeces were separated from the pasture surface, weighed, and stored at −18°C for subsequent determination of the dry weight and the C and nitrogen (N) concentrations after oven drying at 55°C for 72 h.

The C and N concentrations were obtained by the dry combustion method (975°C) using a Vario EL II element analyzer (Elementar Analysensysteme GmbH, Hanau, Germany), detecting C in the form of CO_2_. The limits of detection of C and N were 0.4 *μ*g and 1 mg, respectively.

### 2.2. Greenhouse Experiment

#### 2.2.1. Greenhouse Experimental Conditions

The experiment was performed under greenhouse conditions with the soil collected from the pasture field described above before conducting the pasture field experiment. The experiment was arranged under a completely randomized design in a factorial scheme (6 × 5) with six concentrations of MOX (0, 75, 300, 600, 1500, and 3000 ng·g^−1^ of dry faeces), which were mixed with the soil, resulting in concentrations of 0.0, 1.9, 7.5, 15.0, 37.0, and 75.0 ng MOX g^−1^ of dry soil, five evaluation times (0, 7, 14, 21, and 28 days), and six replicates. The experimental units were 5 L polyethylene pots containing an average of 3,176.8 ± 97.7 g of soil.

**Figure 2:**
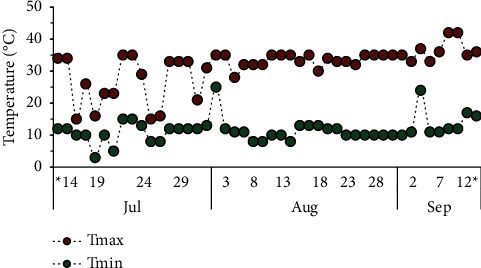
Daily maximum and minimum temperatures (°C) in the greenhouse during the experimental period of the pot experiments, in which moxidectin was incubated in the soil. ^*∗*^The beginning and end of experiment.

The minimum and maximum temperatures in the greenhouse were monitored, ranging on average from 11.5 ± 3.8°C to 31.5 ± 6.6°C, respectively (Figure 2). The loss of moisture from the pots due to evaporation was controlled by weighing the pots, and distilled water was added to rewet the soil as needed.

#### 2.2.2. Animal Faeces

Five Suffolk and White Dorper rams with an average weight of 115.95 ± 14.82 kg and daily faecal production of 3.63 ± 0.80 kg were kept in slatted sheepfolds without receiving any drug treatment for 10 months. They were fed a diet composed of 44.3% concentrate and 55.7% roughage (ryegrass (*Lolium multiflorum* Lam.) hay), following the nutrient requirements established by the NRC [31]. The diet was provided twice a day *ad libitum,* with a 10% daily leftover not to limit feed intake. Canvas bags were used for the total collection of faeces for 24 h.

The amount of faeces collected in 24 h was oven dried at 65°C for 72 h, ground, sieved through a 2 mm sieve, and stored in polyethylene plastic bags in the dark. The samples contained 33% dry matter, 1.7% N, and 37.9% C on a DM basis. The C and N analyses were performed using the Vario EL II element analyzer (Elementar Analysensysteme GmbH, Hanau, Germany).

#### 2.2.3. Moxidectin Treatment of Drug-Free Faeces

Soil moisture and faecal moisture were adjusted to 40% of the water retention capacity (WRC) to ensure maximum microbial activity during incubation. The specific MOX solution for each treatment was added to 80 g of dry faeces, along with the deionized water used to raise the WRC, and subsequently mixed with the soil, considering that 1 mL of Cydectin® contains 10,000 *µ*g of MOX. After homogenization, the experimental units (soil + faeces, with or without MOX) were placed inside the pots and taken to the experiment in the greenhouse.

#### 2.2.4. Evaluation of Soil Microbiota

The evaluation of the population density of soil microorganisms was performed in soil samples that were taken every seven days until the 28th day of incubation. The determination of soil microbial biomass and microbial respiration variables was performed on soil samples taken on the 56th day of incubation.

#### 2.2.5. Cultivable Microorganisms

The soil bacteria, actinobacteria, and fungal colony forming units (CFU) in the soil were determined using the serial dilution technique and Petri dish counting. The first dilution was made with 10 g of soil suspended in 90 mL of autoclaved saline solution. The suspension was stirred at 250 rpm for 15 min in a circular-motion mechanical stirrer. From this suspension, serial dilutions were performed, and 0.1 mL dilutions of 10^−4^, 10^−5^, and 10^−6^ were spread with a Drigalski loop on Petri dishes containing either Thorton culture medium [32] or caseinate-dextrose agar [33] to count the number of colony forming units (CFU g^−1^) of bacteria and actinobacteria, respectively. In addition, 0.1 mL of dilutions 10^−2^, 10^−3^, and 10^−4^ was spread on Martin culture medium [34] to count the number of colony forming units (CFU g^−1^) of fungi. Petri dishes inoculated with the soil dilutions were incubated for seven days at 25°C.

#### 2.2.6. Soil Basal Respiration

The soil basal respiration (BR) was determined in a static system without aeration, according to Alef [35]. Thirty grams of dry soil samples were incubated in 1 L polyethylene flasks, hermetically sealed, in the presence of standardized 0.5 M NaOH, and kept in an oven at 25°C. After seven days, the base excess was titrated with 0.5 M HCl.

#### 2.2.7. Soil Microbial Biomass

Soil microbial biomass was determined by the substrate-induced respiration method [36]. Initially, increasing amounts of glucose (30, 60, 120, 180, and 300 mg) were added to the soil and incubated for 1, 2, 3, 4, and 5 h at 22°C. The stabilization of CO_2_ release was obtained with a dose of 300 mg glucose and an incubation time of 4 h. The results under the conditions of stabilization were used to determine soil microbial biomass with the equation *B* = 40.04 *X* + 0.37, where *B* is the microbial biomass (*µ*g C g^−1^ soil) and *X* is the respiration rate (*µ*g C h^−1^·g^−1^).

#### 2.2.8. Nitrogen (N) Mineralization

After incubating the soil for 56 days, a sample of 50 g of soil per pot per treatment was collected to determine the ammonium and nitrate contents. A total of 150 mL of 1 N KCl was used for the extraction of nitrate and ammonium for the subsequent steam semi-micro Kjeldahl distillation method [37].

### 2.3. Statistical Analyses

The insect's data from the field experiment were analyzed by the Kruskal–Wallis test using the statistical software R (version 2.12.1).

The dry weight and percentages of C and N of faecal boluses were analyzed according to the model: *Y* = *μ* + *B*_*i*_ + *T*_*j*_ + *P*_*k*_ + *T*_*j*_ × *C*_*k*_ + *e*_ijk_, where *μ* = overall mean; *B*_*i*_ = block effect (*i* = 1 to 5), *T*_*j*_ = treatment effect (*j* = 1 to 2), *P*_*k*_ = effect of faeces protection against rain (*j* = 1 to 2, with or without protection against rain), *T*_*j*_ × *P*_*k*_ = interaction between treatment and protection against rain, and *e*_ijk_ = experimental error. The differences were declared significant at 5% probability and the means were compared by a Tukey test.

The CFU results were analyzed considering a completely randomized design with the MOX concentrations and days as factors and using the MIXED statistical procedure of SAS. In addition, polynomial regression analysis determined by linear, quadratic, and cubic contrasts was performed. The results of soil basal respiration and microbial biomass were transformed into Log10 *x*^1.5^ and square root (√), respectively, because they did not have a distribution as determined by the Shapiro–Wilk test. However, arithmetic means of untransformed data are presented.

## 3. Results

### 3.1. Pasture Field Experiment

#### 3.1.1. Coprophagous Fauna

A total of 4,245 Diptera were caught, of which 1,696 were caught in the faeces of nontreated animals, and 2,739 were caught in the faeces from MOX-treated animals, with the method of sweep net and pitfall until 88 days of exposure in the pasture field. The family Sphaeroceridae was the most abundant, with 3,897 individuals (1,482 in the control and 2,415 in the faeces with MOX, with 1,007 and 1,932 in the first ten days, respectively). Muscidae, Sarcophagidae, Sepsidae, and Chloropidae were other frequently identified families (Table 1). Anthomyzidae, Tachinidae, Platystomatidae, Ephydridae, Lauxaniidae, and Chironomidae were caught less frequently by the sweep net method (Table 1), and Sepsidae, Chironomidae, Lauxaniidae, Syrphidae, Phoridae, Tipulidae, Mycetophilidae, and Ephydridae were caught using *pitfall* traps. MOX did not reduce the number of individuals of the order Diptera (*p* > 0.05), nor did it reduce the number of individuals (*p* > 0.05) within each identified family.

A total of 251 and 327 ants were caught in faecal boluses which were observed up to 88 days of exposure in the pasture field for the nontreated and MOX-treated animal faeces, respectively. The genera *Pheidole*, *Dorymyrmex*, and *Acromyrmex* were the most abundant. *Pheidole*, mainly *Dorymyrmex*, *Acromyrmex*, *Camponotus*, *Linepithema*, *Pachycondyla*, and *Brachymyrmex*, were the genera with the highest number of individuals caught by the pitfall method (Table 1). Other genera, such as *Dolichoderus*, *Azteca*, *Hypoponera*, *Pseudomyrmex*, *Solenopsis*, and *Nylanderia*, were present in lower quantities. In the direct counting, the largest number of individuals of the genera *Pheidole*, *Acromyrmex*, *Solenopsis*, and *Hypoponera* was found. For *Pheidole* and *Solenopsis*, the presence of anthills was observed in the faecal bolus (Table 1). *Camponotus*, *Linepithema*, *Brachymyrmex*, *Nylanderia*, *Azteca*, and *Dorymyrmex* were found in smaller quantities. There was no significant effect (*p* > 0.05) in the Formicidae family due to the presence of MOX in sheep faeces (Table 1).

A total of 26 Coleoptera, 20 from the control group and 6 from the MOX treatment group, were observed during the experiment, with *Ataenius* and *Labarrus pseudolividus* being the most abundant. The genera *Onthophagus*, *Ontherus*, *Canthon*, *Dichotomius*, and *Iarupea* were also found, but in smaller numbers. Considering the most abundant Coleoptera, there was no effect due to the presence of MOX (*p* > 0.05) in the sheep faeces (Table 1).

#### 3.1.2. Faecal Degradation in the Field

Faeces C and N concentrations, C : N ratio, and dry weight were affected by treatments (*p* < 0.05) except for faeces dry weight that was not affected by exposition to rain (*p* > 0.05); however, there was no interaction between treatments and exposition to rain (Table 2). The degradation of faeces not exposed to rain decreased (*p*=0.007), since its dry weight was higher compared to faeces exposed to rain. The presence of MOX decreased the N content in the faecal bolus. Consequently, the C : N ratio of faeces without MOX was higher than faeces with MOX (Table 2).

### 3.2. Greenhouse Experiment

#### 3.2.1. Colony Forming Units of Soil Bacteria, Actinobacteria, and Fungi

The population density of microorganisms (bacteria, actinobacteria, and fungi) was not affected (*p* > 0.05) by increasing the concentrations of MOX up to 75 ng·g^−1^ soil (Table 3). There was variation between the periods (*p* < 0.01) without an interaction (*p* > 0.05) between treatments and periods (Table 3). The population densities of the microorganisms were higher at the time of incubation, decreasing with time (Table 3). However, at 28 days, the population density of fungi increased (Table 3).

#### 3.2.2. Soil Microbial Biomass, Basal Respiration, and N Mineralization

**Figure 3:**
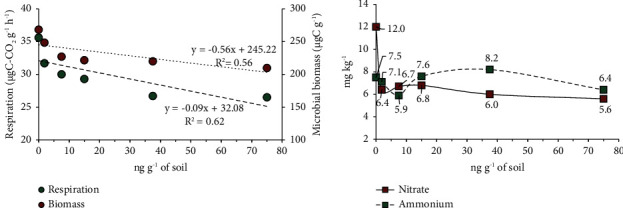
Microbial respiration and biomass in soils after adding faeces of sheep treated with increasing levels of moxidectin (ng·g^−1^ of soil) (a). Concentration of ammonium and nitrate in soils after adding sheep faeces spiked with increasing levels of moxidectin (b).

The treatment of faeces with MOX decreased the soil microbial biomass linearly from an MOX concentration of 1.9 ng·g^−1^ up to 75 ng·g^−1^ (*p* < 0.10; Figure 3(a)). The activity of the microorganisms was impaired by the presence of the MOX residue, since the microbial respiration in *µ*g C-CO_2_ g^−1^·h^−1^ decreased linearly (*p* < 0.01; Figure 3(a)).

The ammonium concentration in the soil solution of samples incubated for 56 days varied from 6.4 to 8.2 mg·kg^−1^, and it was not affected by the addition of MOX (*p* > 0.05; Figure 3). However, the concentration of nitrate in the soil solution decreased by 50%, varying from 11.97 mg·kg^−1^ to 6.34 mg·kg^−1^ in samples contaminated with MOX (*p* < 0.05; Figure 3(b)).

## 4. Discussion

### 4.1. Pasture Field Experiment

#### 4.1.1. Effects of Moxidectin-Treated Animal Faeces on Coprophagous Insects

The results on coprophagous insects obtained in faecal boluses from MOX-treated animals indicated that MOX applied to rams, in a single dosing and according to the manufacturer's recommendation, has no toxic effect on insects that were captured in faeces (Table 1). Notwithstanding, other studies have shown that ivermectin and MOX applied to cattle can decrease the number of adult Coleoptera, *Aphodius,* and *Scarabaeidae* that colonize faeces [14]; that faeces from cattle treated with doramectin, eprinomectin, and ivermectin have reduced insect activity, with lower and confounding effect of MOX [15]; and that MOX may be toxic to coprophagous insects on the first day of administration to equine [38]. On the other hand, it is important to consider that among all the mentioned veterinary drugs, MOX had the lowest adverse effects on insects [15, 20], which may explain the results found in this study (Table 1). Moreover, it was also reported that the effects on insects recovered from faeces depend on the time of exposure in the environment, the route of application to the cattle (subcutaneous or “pour-on”), and the drugs (MOX or doramectin) [20, 39]. Thus, it is possible that, in the studies reported, the dynamics of the coprophagous insect community in the pasture were determined by the variation in environmental factors over time, instead of the MOX applied to the animals.

For example, insects are attracted to faeces by physical factors such as odor, color, and shape. Fresh faeces are colonized almost immediately by adult Diptera, which feed, reproduce, and lay eggs, producing a new generation every 2 to 3 weeks. The number of Diptera declines rapidly after a few hours due to odor reduction [42]. With the method applied in this study, it was not possible to count the number of larvae. However, the number of adult Diptera decreased only after the 10th day (data not shown). In general, faecal colonization is influenced by a succession of events related to temperature and humidity and the effect of such parameters on the desiccation rate [39]. Insect activity tends to be higher in hot and/or humid environments [12]. Interestingly, after 15 days of exposure to the environment (first week of April 2012, Figure 1), the minimum temperature increased, and the increase in temperature may have favored insect growth and faecal attractiveness.

There was a low number of Coleoptera in the faeces, probably because Diptera larvae were not present as well (Table 1). In fact, Diptera are rapidly attracted to fresh faeces, where they lay eggs, which develop into larvae, which in turn attract Coleoptera predators. However, Diptera individuals tend to dominate the colonization of dungs within the first 10 days, while dominant colonization by Coleoptera beetles often occurs after two to three weeks or months [41]. The absence of Diptera and the delay in Coleoptera reproduction explain the low number of Coleoptera in this study (Table 1).

Although the total number of coprophagous insects did not significantly differ between the faeces of MOX-treated and nontreated animals (Table 1), faeces from MOX-treated animals attracted 36% more coprophagous insects. These results corroborate the data reported by Wardhaugh and Mahon [42] who noted that Coleoptera were more attracted to the faeces that contained ivermectin residues (∼61%) compared to the control (∼39%). However, the results from this experiment, indicating that insects are more attracted to faeces from MOX-treated animals, are not corroborated by similar studies with ivermectin elsewhere [43].

#### 4.1.2. Degradation of Faeces under Field Conditions

The degradation of the faecal bolus was more pronounced in the faecal boluses from MOX-treated animals and when they were not protected against rain (Table 2), suggesting that MOX may not be toxic to saprophytic soil microorganisms under natural conditions. The growth and activity of soil saprophytic microorganisms are limited by C sources, soil physical and chemical attributes, physical conditions (temperature, aeration, and humidity), and many types of ecological interactions [27, 44, 45]. Thus, these aspects may interfere with their resistance to xenobiotic compounds. For example, it may be that MOX was, at least, partially metabolized by the treated animals before it was released through the faeces [24]. In that case, MOX was converted into a soluble source of C rather than being a biocide to the soil microbial community, and faeces from MOX-treated animals stimulated soil microbial activity and faecal degradation.

Moreover, the concentration of N in the faecal bolus of MOX-treated animals was higher than that in the faecal bolus of nontreated animals (Table 2). MOX may have killed endogenous parasites in sheep, releasing their N-rich necromass together with blood cells from the mucous region of the digestive system through animal faeces [6, 46]. Likewise, due to C limitation, soil microbial growth is also limited by N supply; for example, changes in the availability of N for microorganisms may impact the diversity of the soil microbial community [47]. Consequently, faecal boluses of MOX-treated animals may have been degraded in different ways compared to faeces of nontreated animals [41] because more N was available to soil microorganisms. However, it should be considered that, in this study, evaluation was made after an MOX single-dose administration, and this cannot be extrapolated to a situation where MOX (or other avermectins) is administered in multiple doses to livestock.

### 4.2. Greenhouse Experiment

#### 4.2.1. Effects of Moxidectin Addition to Drug-Free Faeces on Cultivable Microorganisms

While less than 1% of soil microorganisms are possibly cultivated [48], the cultivation of the most abundant microbes may demonstrate the effects of MOX on cultivable soil microorganisms. The CFU counts of bacteria and actinobacteria were higher at the time of incubation and decreased over time until the 28^th^ day (Table 3). This can be attributed to the availability of nutrients with the inclusion of manure and gradual depletion, since a single fertilization was performed at the beginning of the experiment. The counting of fungal CFUs showed a significant increase at 28 days after the inclusion of faeces, regardless of the presence or absence of MOX residue, as the temperature increased. Thus, the abundance of CFUs of the microorganisms was affected by environmental conditions, especially by higher temperature (Figure 2) and the availability of substrate in the soil.

On the other hand, the presence of MOX in the faeces did not affect the CFU of fungi and bacteria in the growth media (Table 3). Macrocyclic lactones, such as MOX, are considered effective against helminths and may affect insects and arachnids, but they are considered inactive against annelids, bacteria, and fungi [23, 25, 49, 50]. An early study showed that concentrations of 10^−8^ to 10^−3^ M of pure ivermectin in growth broth affected the reproduction and development of some filamentous fungi but not others [49]. A few years later, Lim et al. [23] examined the antibacterial effect of four avermectins (doramectin, ivermectin, MOX, and selamectin) and found no inhibitory effect on the bacteria *Escherichia coli*, *Acinetobacter baumannii*, *Pseudomonas aeruginosa*, *Streptomyces lividans*, *Kocuria rhizophila*, and *Staphylococcus aureus*. However, there was reported evidence that MOX may inhibit the growth of *Mycobacterium tuberculosis* [23] and *M. ulcerans* [51] which is desirable, but not of other rapidly growing mycobacteria [50]. Our results indicate that MOX is inactive against cultivable soil microorganisms.

#### 4.2.2. Soil Microbial Biomass and N Mineralization

MOX at concentrations varying from 1.9 to 75 ng·g^−1^ soil (at 40% moisture) had a detrimental effect on microorganisms, as respiration and microbial biomass decreased linearly, and nitrification decreased by 50% with the inclusion of MOX, regardless of the amount used (Figure 3(a)). These results corroborate the conclusions reported by Ritz [48] that controlled environmental conditions with microbial pure cultures may not always be applied to microbes living in soil conditions.

Moreover, the methodology of soil microbial biomass measures the C contents of all soil organisms smaller than 5 × 10^3^ *μ*m^3^, such as archaea, bacteria, fungi, protists [36, 52], and even other eukaryotes, such as nematodes measuring small sizes [53], as long as they pass through the sieve during soil preparation for incubation. It is possible that several prokaryotes, including ammonia-oxidizing archaea and bacteria, which oxidize ammonia to nitrite, are involved in the process of NH_4_ oxidation [54] and are negatively affected by MOX in the soil. These microorganisms were probably not isolated with methods that were used to produce the results in Table 3.

Considering that both ivermectin and MOX are macrocyclic lactones, our study can be compared to that reported by Halley et al. [24]. By incubating two different sandy loam soils (pasture and forest) with faeces obtained from steers treated with ivermectin (resulting in a nominal concentration of 30 *µ*g ivermectin g^−1^ dry weight soil) for 30 days, Halley et al. [24] demonstrated that ivermectin administered to animals and eliminated in the faeces did not affect soil respiration and ammonia utilization (nitrification). Additionally, Halley et al. [24] highlighted that the concentration of the total faecal residue (sum of ivermectin and its metabolites) of steers treated with ivermectin in the soil was 0.2 ng·g^−1^ soil, containing 0.09 ng·g^−1^ unmodified ivermectin, and they concluded that it would be very difficult that application of ivermectin to animals would impact soil microorganisms. The conclusions made by Halley et al. [24] agree with the results from our field experiment, in which we tested faeces excreted by MOX-treated sheep.

In general, Brazilian sheep farmers use MOX in 30-day intervals or less, which is considered a high-frequency preventive method [7]. However, there is a practice of deworming at higher doses than those recommended by the manufacturer [55]. This is a direct consequence of the low efficacy of some drugs, and in a vicious cycle, selection of resistant parasite populations has been observed. In addition, this drug use regime also imposes risks of environmental contamination [7, 56]. In this regard, the results obtained under greenhouse conditions showed adverse effects on microbial activity in the soil (by determining the degradation of dry matter in faeces and the concentration of N and C) (Figure 3) and on microbial respiration and biomass due to the presence of MOX in sheep faeces, even at the lowest MOX concentration studied (75 ng·g^−1^ faeces, corresponding to 1.9 ng·g^−1^ of soil) (Figure 3). In a previous study, we reported MOX residues of approximately 30–35 ng·g^−1^ in sheep faeces in subtropical pastures [10]. However, Sanhueza [57] reported MOX concentrations up to 350 ng·g^−1^ in sheep faeces under field conditions. Therefore, considering the importance that soil microbial biomass has on soil nutrient cycling [27, 42, 58], we cannot exclude the possibility that MOX contamination can affect soil microbial biomass and soil nutrient cycling mainly when MOX is present in faeces in greater amounts than it is excreted when sheep are farmed under good veterinary practices.

## 5. Conclusions

Faeces from sheep that received a single subcutaneous dose of 0.2 mg·kg^−1^ body weight of MOX are not harmful to the coprophagous insects. In fact, these faeces are more prone to degradation in the environment. However, when faeces excreted by MOX-treated sheep resulted in soil MOX levels higher than 1.9 ng·g^−1^, this decreased soil microbial biomass and N mineralization, probably by affecting the growth of noncultivable microorganisms. These results should be considered in the establishment of guidelines for the environmentally safe use of MOX in sheep livestock.

## Figures and Tables

**Table 1:** Number of coprophagous insects in the faeces of sheep treated with moxidectin, with or without protection against rain in the field pasture, using the sweep net, pitfall, and direct counting methods, obtained in field experiments performed in Pinhais, PR, Brazil.

Coprophagous insect	Treatments				*p* value^(1)^
	Control without protection	Moxidectin without protection	Control with protection	Moxidectin with protection	
*Diptera/sweep net*					
Sphaeroceridae	145	228	74	137	0.13
Sarcophagidae	4	5	2	3	0.47
Muscidae	4	8	2	7	0.05
Chloropidae	2	2	1	2	0.62
Ulidiidae	1	2	1	1	0.57
Sepsidae	0	4	2	2	0.02
Bibionidae	0	0	1	1	0.09
Dolichopodidae	2	2	1	0	0.13
Phoridae	0	0	1	0	0.19
Syrphidae	2	1	0	2	0.19

*Diptera/pitfall*					
Sphaeroceridae	3	6	5	5	0.64
Muscidae	1	2	1	2	0.85
Sarcophagidae	1	2	0	1	0.76
Dolichopodidae	0	0	0	1	0.07
Bibionidae	1	0	0	0	0.14
Chloropidae	1	1	2	1	0.78
Sciaridae	0	1	1	1	0.42
Cecidomyiidae	0	0	1	1	0.71
Ulidiidae	0	0	0	1	0.11

*Hymenoptera and Isoptera/pitfall*					
*Pheidole* sp.	8	7	9	10	0.41
*Dorymyrmex* sp.	1	6	4	5	0.05
*Camponotus* sp.	0	1	1	1	0.72
*Linepithema* sp.	1	2	2	2	0.79
*Acromyrmex* sp.	1	3	2	2	0.27
*Pachycondyla* sp.	2	1	1	1	0.47
*Brachymyrmex* sp.	1	1	1	1	0.76
Other	2	4	3	3	0.43

*Hymenoptera and Isoptera/direct counting*					
*Pheidole* sp.	1	5	4	8	0.78
*Acromyrmex* sp.	3	3	5	3	0.34
*Solenopsis saevissima*	3	0	0	1	0.51
*Hypoponera* sp.	0	1	2	1	0.10
Termitidae	0	0	0	2	0.57

*Coleoptera/pitfall and direct counting*					
Scarabaeidae: *Ataenius* sp.	2	0	1	0	0.19
Aphodiidae: *Labarrus pseudolividus*	1	0	0	0	0.05

^(1)^ *p* value determined by the Kruskal–Wallis test.

**Table 2:** N and C concentrations (%), C : N ratio, and dry weight (g) of faecal boluses of nontreated (control) and moxidectin-treated (MOX) animals in the field pasture, with or without protection against rain, in field experiment performed in Pinhais, PR, Brazil.

	Treatment (T)		Protection against rain (P)		*p* value		
	Control	MOX	Without	With	*T*	*P*	*T* × *P*
C (%)	36.3^b(1)^	37.3^a^	37.0	36.7	0.038	0.578	0.437
N (%)	1.46^b^	1.71^a^	1.49	1.69	<0.001	0.742	0.303
C : N ratio	25^a^	22^b^	24	24	<0.001	0.987	0.522
Dry weight (g)	51.1^a^	37.5^b^	41.8^b^	48.6^a^	<0.001	0.007	0.966

^(1)^Means followed by different lowercase letters in the same row of the treatment columns differ (*p* < 0.05) by Tukey's test. Table shows that regardless of protection against rain, the MOX treatment had a notable impact on the levels of C, N, C : N ratio, and dry weight in faecal boluses. In contrast, the presence of rain or its interaction with treatments did not show statistical significance.

**Table 3:** Colony forming units (CFU g^−1^ soil) of bacteria, actinobacteria, and fungi in soil with dried faeces treated with moxidectin, responding to increasing concentrations of MOX in the faeces over time, in pot experiments performed under greenhouse conditions.

Microbe	MOX concentrations (ng g^−1^ soil)						Period of incubation (days)^(1)^					*p* value^(2)^		
	0	1.9	7.5	15	37	75	0	7	14	21	28	*T*	*P*	*T* × *P*
Bacteria (×10^8^)	2.90	2.70	2.68	2.85	2.42	3.14	6.21a	2.38b	2.44b	1.38c	1.43c	0.161	<0.01	0.992
Actinobacteria (×10^8^)	2.26	2.92	3.33	2.68	2.22	3.19	6.52a	2.10b	2.25b	1.66bc	1.40c	0.101	<0.01	0.963
Fungi (×10^4^)	3.26	3.25	4.22	2.13	2.06	2.80	1.80b	1.50bc	1.25c	1.78b	9.61a	0.744	<0.01	0.997

^(1)^Means followed by different lowercase letters in the same row within the same category differ (*p* < 0.05) by Tukey's test. ^(2)^*T*: *p* value for the effects of treatment in the orthogonal test. *P*: *p* value for the effect of periods. *T* × *P*: *p* values indicate that there was no effect of the interaction between treatments and periods. Therefore, only the means due to individual factors (MOX concentrations or period of incubation) are shown.

## Data Availability

Data are available on request.

## Additional Points

*Highlights*. (i) Sheep treated with moxidectin excrete faeces that contain residues of the drug. (ii) Moxidectin-treated sheep faeces may harm soil biodiversity. (iii) Moxidectin-amended faeces from drug-free sheep lowered soil microbial activity.
